# Health through the eyes of youths: a qualitative study

**DOI:** 10.3389/fpubh.2024.1271215

**Published:** 2024-05-17

**Authors:** Nikki Ow, Rebecca Zivanovic, Karen Tee, Steve Mathias, Skye Pamela Barbic

**Affiliations:** ^1^Department of Occupational Science and Occupational Therapy, University of British Columbia, Vancouver, BC, Canada; ^2^Department of Psychiatry, University of British Columbia, Vancouver, BC, Canada; ^3^Foundry, Vancouver, BC, Canada

**Keywords:** substance use and misuse, psychiatric disorder, health, quality of life, access to health and social services, youth

## Abstract

**Background:**

Emerging definitions of health have suggested a shift in focus to one’s ability to manage their health condition, function, and social determinants of health. The construct of health for youths with mental health and substance use disorders (MHSU) is complex and multi-dimensional with interplay between biological, behavioral, and social conditions. Expanding definitions of health is crucial in the measurement of health and evaluation of integrated youth services (IYS) systems for people with MHSU disorders. Hence, it is critical to understand the construct of health from the perspective of a young person living with a MHSU disorder.

**Methods:**

This study was conducted using inductive thematic analysis. Three focus groups were conducted from July to August 2017.

**Results:**

A total of 22 youths (17–24 years) took part in this study. Results showed that health is a multidimensional construct situated in the ecosystem of a person’s environment. Health can be understood from two macro themes: Individual health and Determinants of health. It consisted of physical health, mental health, day-to-day functioning, and being in control of your own health condition. Systemic and social factors were factors that influenced the state of health.

**Conclusion:**

This study contributes to a conceptualization of good health in youth with MHSU disorders. This conceptualization can aid in the development of more accurate measures of health and functioning and the evaluation of mental health services for youth with MHSU.

## Introduction

According to the World Health Organization (WHO), the definition of health is “*a state of complete physical*, *mental and social well-being and not merely the absence of disease or infirmary*” (1). This definition of health was formulated in 1946 in the post-war era and has not been changed even though the definition of health and the notion of what is considered “healthy” has evolved over time (2). The current definition of health is problematic as it implies that a healthy individual is one who always has complete physical, mental, and social functioning. Few people, if any, can fit this definition of health. This definition also minimizes the role of human capacity to cope and labels those with chronic, progressive, or relapsing–remitting conditions permanently unhealthy (3, 4). These criticisms are specifically relevant in populations with health conditions that are often chronic or relapsing–remitting like mental health and substance use (MHSU) disorders.

Emerging definitions of health have suggested a shift in focus to one’s ability to adapt and self-manage (3), social determinants of health as well as policy and environment (4, 5), and functionality and wellbeing (6). A new definition of health as “the ability to adapt and to self-manage” has been proposed (3). This definition includes the one’s ability of people to adapt to their life situation and acknowledges that health differs from person to person. This definition, however, makes it difficult to measure health on a population level.

Expanding definitions of health is crucial in understanding the impact of MHSU disorders on health and in designing health services for people with MHSU disorders. Mental health is vitally important to overall health (7). This is especially important in adolescence and young adulthood where MHSU disorders are disproportionately more prevalent in youths aged 15–24 (8–10). The onset of many other mental health conditions occurs during this period of development (9, 11) (p. 8) and often the use of substance use further exacerbates the mental health condition (12) (p. 423). In addition, studies have shown that youths with substance use disorders have increased risk of developing psychiatric symptoms and disorders (13, 14). MHSU disorders in adolescence also increases the risk of comorbid physical health problems in later life (15–18) as well as adverse social outcomes in adulthood such as lower educational attainment, poverty, and social isolation (19). It is therefore critical for the healthcare system to engage youth early and intervene early (20)

Youth with MHSU disorders experience multiple health and social challenges and therefore require comprehensive interventions. Integrated youth services (IYS) models are being proposed to support the full range of health and social needs for youth (21–23). The IYS model aims to improve the quality of mental health and substance use services for youth by providing multiple services in youth-specific settings (24, 25). In IYS models, youth are crucial stakeholders in designing how health services are delivered and evaluated. However, there is still a significant gap in understanding these health needs and priorities, as defined by youths themselves (26). A critical step toward this is establishing a unified understanding of how “health” is defined and conceptualized from the perspective of young people. Formal definitions and current frameworks of health might not be sufficient to explain the perspectives of youth with MHSU conditions.

To build a youth-centered healthcare system for youths with MHSU disorders, it is critical to understand and identify important health outcomes that matter to youth. Therefore, the central question of this study is “What is the concept of good health in youths who experienced MHSU disorders?” The objectives of this study were to understand and identify component of good health through the perspectives of youths who have experienced MHSU disorders and identify important factors that affect their ability to achieve good health. Results of this qualitative study will allow us to understand important prognostic factors and identify important outcomes that should be measured in a IYS system.

### Participants and recruitment

Participants were recruited using purposeful sampling. Flyers were handed out by staff and posted in the common areas of an IYS center providing health and wellness resources, services and supports to youth with MHSU disorders in (blinded for review). Clients at this center can self-refer or be referred to services by a health practitioner. Services at this community-based center were delivered by a team of interdisciplinary healthcare professionals (physicians, nurses, counselors, and peer support specialists). Eligibility criteria included youths who have received or are receiving MHSU services, aged 17–24 years, willingness, and ability to read and respond in English, and able to provide informed consent or have a guardian to provide consent. This age group was chosen because they were in a developmental transition phase (i.e., from high school to college; college to work). As access to specialized mental healthcare is scarce for this vulnerable population our research time decided that a formal diagnosis of MHSU disorders was not required for study participation. All participants provided written informed consent prior to the interviews. Participants below 19 years of age provided informed assent to participate in the focus groups and had a guardian who gave consent. A small honorarium was paid to all participants. This study was approved by the (blinded for review).

### Data collection

Three focus groups were conducted between July to August 2017 at the same center where participants were recruited and accessed their mental health services and, hence, was perceived as a safe and inclusive environment. Focus group technique was chosen because the aim of this study was to build a better understanding of the construct of health. There is existing knowledge of the construct of health, but the current framework is inadequate when used in mental health conditions. The use of focus groups allowed us to generate more themes (27). We stopped data collection when data saturation was reached; that is, if similar codes and meaning of codes were repeated in subsequent interviews (28).

Each focus group lasted approximately 90 min. All participants in the focus groups were grouped by age to encourage interaction (two groups ages 20–24 years, one group ages 17–18 years). Three main questions were asked in the focus groups:

1. What does health mean to you?
2. What does it look like to be in a good/poor health state?
3. What is needed for someone to move from a poor to a good health state?

All focus groups had two facilitators. The primary facilitator was a research assistant with qualitative research methodology experience. This facilitator had limited, if any, exposure to participants prior to the focus groups. The other facilitator was the study’s peer research partner who was also a peer support worker (PSW) at the center. A PSW in our context is an individual with lived experience of mental health and/or substance use issues providing support based on this shared experience. The PSW did have prior relationships with some participants of the focus group and was an established presence in the center. During the focus groups, this facilitator mainly wrote field notes and provided some prompts. Also in the room was the last author who observed and took field notes. All interviews were recorded and transcribed verbatim by the second author and a research assistant.

### Data analysis

Data analysis was conducted on NVivo 10. Transcripts were de-identified before being uploaded onto NVivo. Data were analyzed using thematic analysis utilizing an inductive approach. This approach allows researchers to systematically understand the experiences of groups who have navigated or are still navigating the ups and downs of the recovery process and explore what achieving “good health” mean for them (29). The first two authors performed the analysis. Transcripts were read through once before coding. Thematic analysis utilizing a data-driven, inductive approach was used to analyze the data (27). At the second reading, notes were written and data were organized into broad thematic codes. Multiple readings of the transcripts allowed the researchers to focus on the subjective experiences of the participants and practice reflexivity. A preliminary list of codes was formed from the first transcript. Coding was performed after each interview. This process was repeated for the next two transcripts. New codes that emerged from each transcript were added to the list. A saturation grid was created to reflect the common codes in each focus group. After going through the third transcript, no new codes were added. All codes from each focus group were then analyzed at the micro level to inductively identify overarching themes. Agreement of final themes was achieved through an iterative process of discussion among research team members and constant review of the data.

Member checking was conducted twice with three of the participants throughout the analysis process. Participants reviewed all themes and agreed that they were understandable and representative. The first time was after broad and micro themes were proposed. Participants reviewed and ensured that all themes were understandable and representative. The second point of contact was to confirm the proposed definition of health and quotes selected were appropriate, understandable, and representative. Data source triangulation was also conducted with the notes from the facilitators and the author present at the interviews (SB).

## Results

### Characteristics of participants

Altogether, 22 participants participated in three focus groups (Table 1). Participants were between ages 17–24 years with a median age of 22.5 years, 12 participants identified as female, and the most of participants identified as White while two identified as Indigenous. All participants have accessed and received services from an IYS center. In terms of employment status and education, 12 participants were unemployed and not in school and 18 participants had a high school diploma. Out of the 22 participants, eight were in at-risk housing situations such as being homeless, couch surfing or living in a single room occupancy hotel. Thirteen participants had used cannabis or other illicit drugs in the past month. As for primary health condition, participants reported mental health diagnoses including anxiety disorders (86%), mood disorders (86%), post-traumatic stress disorder (36%), borderline personality disorder (23%), psychotic disorders (18%), autism (9%), and attention deficit/hyperactivity disorder (5%).

**Table 1:** Characteristics of focus group participants (*n* = 22).

	Number of participants
Median age (age range)	22.5 (17–24)
Group 1 (*n* = 10)	23.1 (21–24)
Group 2 (*n* = 8)	22.4 (20–24)
Group 3 (*n* = 4)	17.8 (17–18)
Gender	
Female	12
Male	7
Non-binary and transgender	3
Ethnicity—white	16
Completed high school	18
Employment status	
In school	4
Employed	3
Not employed or in school	12
In at-risk housing situation	8
Alcohol use > 1x per week	7
Cannabis use > 1x per week	6
Other illicit substance > 1x per week	5

### Construct of health

**Figure 1:**
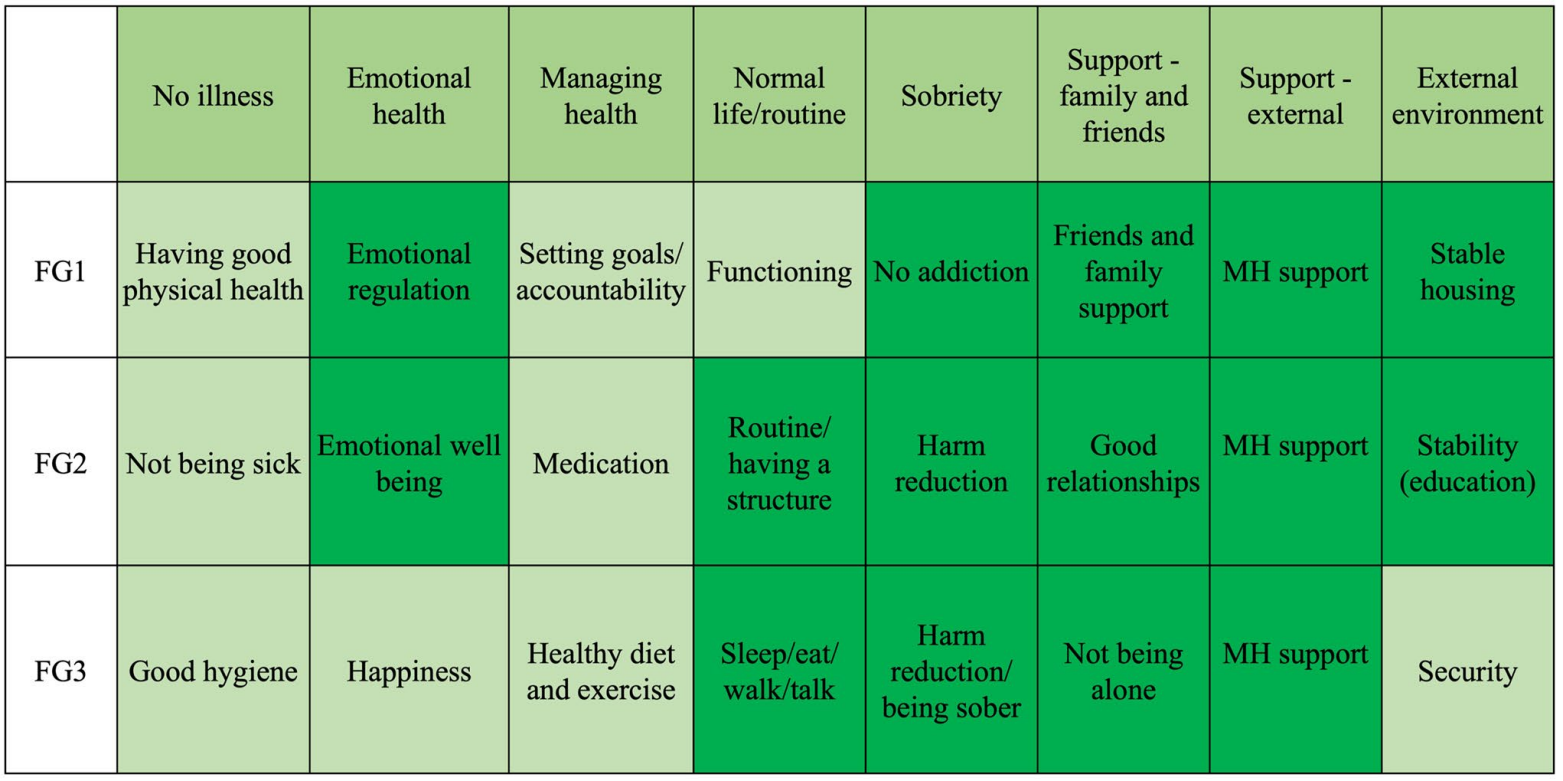
Saturation grid. ^*^The darker the green, the more prominent the theme.

**Figure 2:**
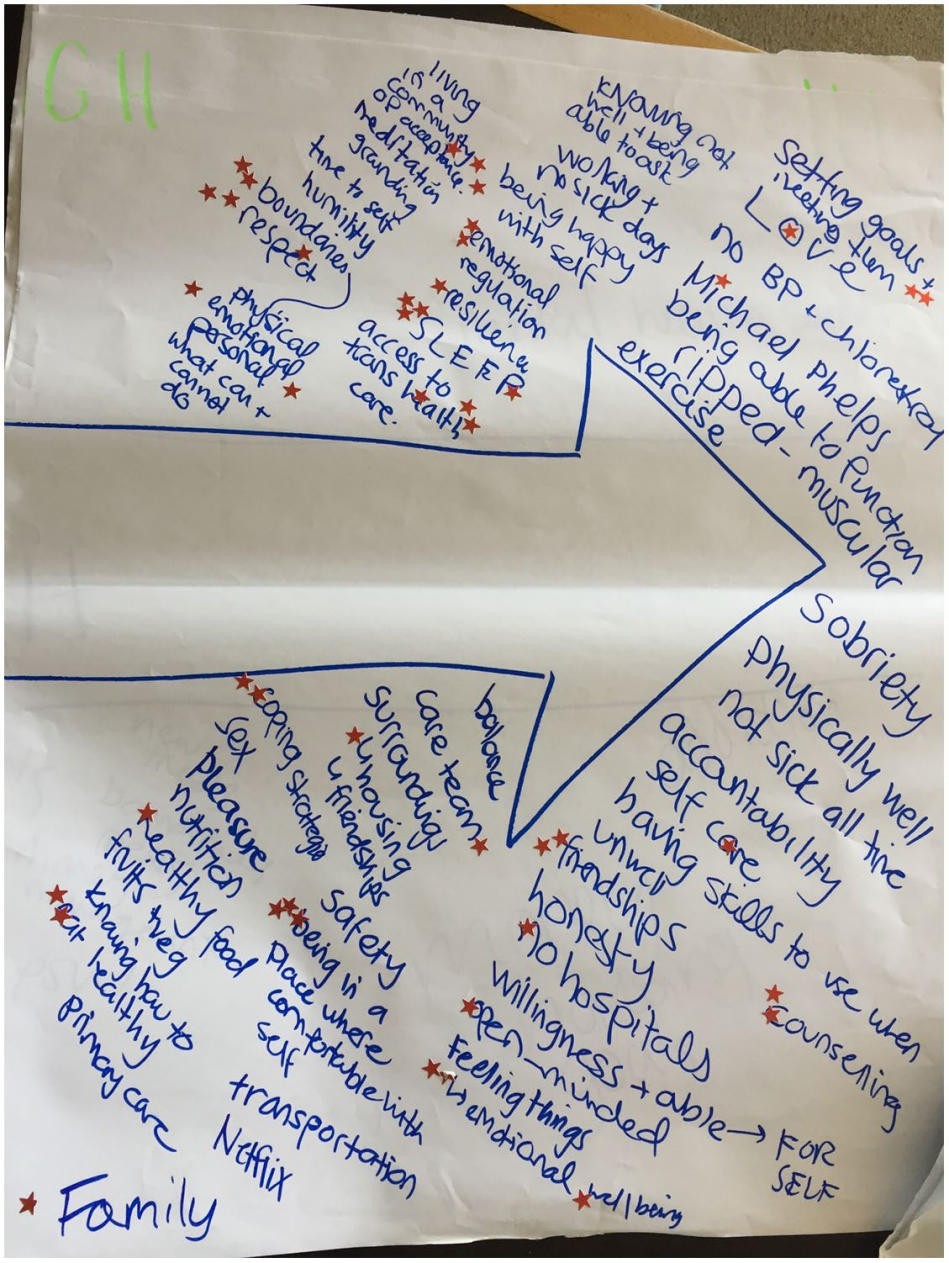
Picture of focus group discussion.

**Figure 3:**
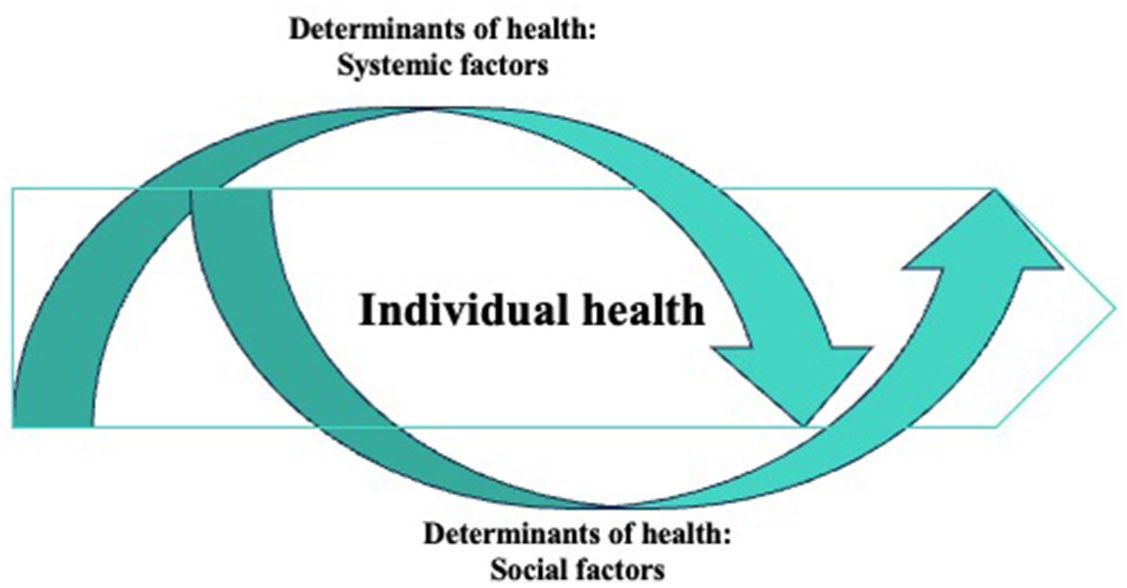
Image illustrating recovery as a continuum and the relationship between themes.

A summary of the themes that emerged from all three focus groups is shown in the saturation grid (Figure 1). In the eyes of our participants, health was a continuum of many personal and environmental factors. These factors were intertwined together, and the concept of good health cannot be separated from its context. Figure 2 shows a description of health drawn by participants in one of the focus groups. This concept can be understood from two macro themes: *Individual health* and *Determinants of health*. The relationship between these two themes is illustrated in Figure 3 where the individual health is a continuum in the whole ecosystem of social and systematic factors that determine health. Additional quotes are provided in Table 2.

**Table 2:** Additional quotes for each subtheme.

Themes	Additional quotes
Individual health	
State of mental and physical wellbeing	“*Maybe steady exercise*, *everything like that*, *to make sure your body functions at a good rate*”*—*FG1.
	“*Eating good food*. *Having a good diet*”*—*FG2.
	“*I’d say people who just like*, *people who are super in shape*, *eating a super good diet*, *like eat really healthy*, *like maintain*, *have steady work*, *get a full amount of sleep*, *everything like that*”*—*FG2.
	“*And healthy activities*, *doing a sufficient amount of healthy activities*. *That you consider fun*, *you know what I mean*. *Doing things that make you happy*”*—*FG3.
	“*Brushing your teeth*, *combing your hair*. *Wiping your ass well after taking a dump*, *who knows*, *you know*”*—*FG3.
Emotional health	“*If you are depressed and feel anxious all the time*, *you are going to have more sickness and more*, *you know*, *diseases than somebody who is therefore not*, *for the simple reason that you know*, *I do not know*”*—*FG3.
	“*They do not really*, *like*, *go out to help themselves*. *They kinda just stay in either a depressed state or like an unemployed state*, *they do not really go out to better themselves*”*—*FG2.
	“*Just being happy with yourself*. *Not being frustrated with*, *you know… Not that you are perfect*, *just like not being frustrated with aspects of yourself*”*—*FG1.
	“*Feeling in control of your thoughts and behavior*”*—*FG1.
Day-to-day functioning	“*…structure for me is that when I’m using and stuff like that one thing that makes me continue to use is that I’m bored*, *and if I do not have structure in my life which means having appointments on certain days*, *and making sure after the appointment maybe 30 min later I have something else*, *and then knowing that after dinner I have nothing to do maybe go to a meeting*, *make sure that my day is productive*”*—*FG1.
	“*…structure*, *similar idea I guess*, *um thinking about like*, *in addiction particularly I guess often*, *you know*, *sometimes the addiction itself provides a type of unintentional structure because that’s your thing that you do*, *all day*. *So when all of a sudden you try to stop you now have a totally empty day and you have no idea what to do with it*, *and that makes you wanna fall back*, *so to replace it with some sort of new structure so to just do things*, *just for the sake of having something to do every day*”*—*FG1.
	“*Like*, *um*, *yeah like does an activity*, *has a job*, *goes to school*, *has like all the boxes ticked off*. *One of those*, *yeah*”*—*FG3.
	“*Not taking care of yourself*, *or not being able to*. *And not functioning*, *in anything*”*—*FG3.
Management of health	“*All this is like something that you need to be like… it’s not something that happens overnight*, *like there’s gonna be times when you struggle and stuff like that*, *so I think everything should be ongoing practice*”*—*FG1.
	“*Yeah*, *if you learn how to manage your time better and actually spend your time trying to help yourself and everything like that you’ll move a lot faster instead of just procrastinating*”*—*FG2.
	“*I think it’d be like*, *as what I said before about knowing your body*, *what works for you*, *what does not*. *Ummm*, *knowing… Someone who’s on the high end I do not think they are gonna be like super happy all the time*, *you know*, *knowing how to handle things when things get rough*”*—*FG3.
Determinants of health	
Systematic factors	“*I guess like stability of your necessities like food*, *water*, *and medication*”*—*FG 1.
	“*…not knowing that there was a clinic down here for youth*, *and it is not a place for someone who’s 24*, *25 to be*, *…so I would feel way more comfortable if I was still able to access my services at [name of center]*. *And*, *honestly*, *it gives me anxiety*, *it scares me more*, *and it makes me wanna act out knowing that I might not be here next year or the year after*, *so*, *it feeds my addiction and my mental health*”*—*FG1.
	“*And I do not want that kind of fear*, *I wanna be able to access the kind of stuff*, *all the resources and not feel like I’m gonna be cut off at any moment and knowing-the fear of the unknown scares me and makes me wanna*, *uh*, *sabotage myself*”*—*FG1.
	“*you are stressed out about time management*, *like… talking to a peer support worker that would be able to help you like*, *break down the problem and you’d be able to like get around it easier*”*—*FG2.
	“*Yeah because if something were to go wrong*, *you do not have to worry about* ‘*Oh*, *I cannot afford the dentist*’ *or* ‘*I cannot do whatever*’ *so that’s like*, *less mental stress I guess*, *even though there could be other problems*. *It’s one less thing they have to worry about is like having money*, *or…*”*—*FG3.
Social factors	“*Um*, *when I think of like good health I think of knowing when I’m not well*, *and being able to ask for help at those times*”*—*FG1.
	“*Cause stress can cause like heart attacks*, *and if you do not have good social*, *like*, *like*, *if you are not social and do not have friends then you can get like dementia and stuff*”*—*FG2.
	“*Because*, *you know*, *a healthy group of friends or family is healthy*”*—*FG3.

### Individual health

Participants were asked about good and poor health states and their perception of these states. Conceptualizations of good and poor health were often multi-dimensional and defined by most as being beyond the absence of illness or “*not being sick*.” One common thread that was brought up in all three focus groups is the subjective and individualized nature of the concept of health.

“*It’s really like*, *unique in what it means to you*. *Like being healthy could mean like exercising every day*, *doing as much as you can*. *For some people it might just be having enough energy to leave the house*, *or like—I think it really depends on*, *like what you need and what you consider to be healthy*, *you don’t have to be… live up to some sort of standard I guess*.”

The subthemes illustrating this theme were associated with individual physical and emotional issues and struggles.

#### State of mental and physical wellbeing

When asked what good and poor health looked like, participants related it to their individual experiences with MHSU issues. Many participants felt that good health was associated with achieving “*a state of mental and physical wellbeing*,” “*not… being sick all the time*.” One even compared good health to the swimmer “*Michael Phelps*” because “[he is] *healthy in most ways*.”

A good health state was commonly associated with the concept of “*sobriety*.” Someone in a good health state was described as having “*frequent lengthy periods of sobriety*” as opposed to someone in a poor health state who was described as, “*using drugs*, *using alcohol*, *using any kind of like substances*” and “*every few minutes you need a sip of beer*, *or every hour you need a hoot of crack*.” The consequences of poor mental and physical wellbeing associated with substance use were also described by some participants who struggled with substance use, having “*track marks and abscesses*,” “*scars all over face*” from formication and being “*underweight because of drugs*” were examples of poor health. To them, these physical scars are a reminder that they are not in good health, as one participant articulated, “*health to me is not having really bad track marks on my arm*. *Cause*, *you know*, *if you look at my arm*, *I*, *I’ve used almost every day in a row*, *sometimes I’ll go a few days without using*, *but every time it’s in this arm*, *and I’ve got like*, *you know*, *two baby tracks from yesterday and those’ll be gone tomorrow and I got this one from three days ago that just sorta turned into a zit…But health is*, *you know*, *taking care of my arms and making sure that if I do use drugs I do not have horrible track marks*, *you know*, *it’s um…*.”

#### Emotional health

Emotional health was associated with emotional stability and resiliency without a specific focus on happiness. Good emotional health is “*where you can function and live your daily life and your daily activities*” and “*not falling in the pressure and like anxiety attacks and stuff like that*. *Like more capability to deal with your problems rather than like*, *break down*.” “*Feeling things*, *feeling emotions…the whole range*” was important to a good health state. Many negative emotions like, “*anger*, *sadness*,” “*self-loathing*, *despair*,” “*overthinking*,” “*impulsivity*,” “*suppressing*,” and “*being hateful*” were listed as being examples of a poor emotional health. Having good emotional health also did not mean experiencing only positive emotions all the time, as one participant clarified: “*I do not think they are gonna be like super happy all the time*, *you know*, *knowing how to handle things when things get rough*.” Good emotional health was also associated with “*knowing your personal boundaries and being aware of what your own limits are*.”

#### Day-to-day functioning

This subtheme was described by many participants as being “*able to function*,” or “*not functioning*” on the inverse. When asked what good health was, one participant described it as “*Being able to function the way you want to*.” Good health associated with day-to-day functioning was described as a state “*where you can function and live your daily life and your daily activities*.” Another participant described functioning on a more personal level as “*being able to function the way you want to*.”

Day-to-day functioning was associated with social interaction and routine. Routines and a structure in life were also part of good health. This was explained by one participant, “*like routine*, *um so if you are getting*, *like maintaining steady sleep patterns*, *getting out of the house every day*, *have a good heart rate and stuff like that…*” while another suggested that it involved simply “*getting up in the morning*.”

#### Management of health

Knowing how to manage your own health was a central theme to being in good health. Many participants associated good health as being someone who attends medical appointments and takes their medications. For example, someone in a good health state “*sees a doctor regularly*.” Being accountable with taking medication and attending scheduled appointments was also necessary to maintain good health.

Personal factors were also quoted when participants talked about management of health. Many participants recognized that there were certain qualities or attributes that an individual can foster that will help them in achieving good or better health. One was the importance of “*goals*, *long-term and short-term*” and “*having something to look forward to*.” This was best summarized by this quote: “*because if you do not want to change then you are not going to*.” In addition, participants felt that it was important to be resilent and have “*coping strategies*” so that “*you are able to not be affected by environmental factors of like*, *things that would pull you down*.” Beyond that, “*persistence*” or “*dedication*” was also needed because as one participant puts it, “*it’s not something that just happens overnight*, *like there’s going to be times when you struggle*.” This was echoed by another who said, “*You cannot just rely on medication or counselors*, *you have to put the work in and I think that’s the hardest part*.”

### Determinants of health

The concept of individual health sits in the ecosystem of determinants of health. Participants in our study identified many factors that could affect their health beyond individual efforts. These determinants of health were factors that contributed to their health status. The subthemes under this theme were systemic factors, and social factors.

#### Systemic factors

This subtheme described the external environmental factors associated with health. Access to health services was most frequently quoted by the participants in our study. This included timely and accessible care from “*a health care team*” including family physicians, nurse practitioners, psychiatrists, counselors, social workers, and case managers. This also included access to “*medications*” and “*harm-reduction*” including “*narcan*,” treatment centers, “*youth detox*,” addiction specific counseling and opioid replacement therapy (“*ORT*”). Being able to access these services was essential for good health. However, many participants felt that long waitlists and limited opening hours made it difficult for them to access the help that they needed. Some participants had concerns regarding age limits and found these limits to be arbitrary. They also expressed frustration that accessing services was often disjointed and required going to many places and/or through many people to access services that seemed related. These were factors that they could not control but could lead to a poorer health outcome for them.

Socioeconomic status was another important contributor to health. Many participants on social assistance felt that social assistance payments were too low and that having “*higher pay*” or even a “*higher minimum wage*” would help one achieve a better health state. They also associated being on welfare as not being in good health, because “*somebody on the high end of the scale preferably probably not be on welfare because usually it’s when you are at the… I think you are gonna have excess*.” As another participant put it, “*welfare’s not enough to survive on*, *you are not gonna be thriving in health or food or housing or anything like that surviving on welfare*.”

#### Social factors

This subtheme describes how interpersonal relationships relate to health and includes references to a support system within and outside of a formal health care team. People in a good health state were described as having “*good relationships*” and “*being able to communicate well with others*.” Good support systems were made of “*people that are there for you like all the time*, *like somebody you can always reach and get a hold of*” and included “*family and friends*” and “*other people who have gone through the same thing*,” with specific reference to “*peer support workers*” and “*NA* [narcotics anonymous], *AA* [alcoholics anonymous] *sponsors*.” Having a good support network was also important for good health, “*cause those people will never let you be in a bad state*,” but “*unhealthy friendships*” and isolation were a negative impact on health. This was summarized by one participant who shared: “*isolation*, *you know feeling like you do not have a family or a community or friends*, *whatever that looks like to you*, *but the absence of it is very unhealthy*.” Being alone and isolated was also associated with poor outcomes as reiterated by another participant, “*cause lonely people*, *kinda die*. *Die off*.”

Help to access services such as employment, housing and education was also highlighted. Searching for resources was often confusing and difficult; having access to people who could help them navigate pursuing education in the context of mental health disorders would be helpful. Other factors having a positive impact on health that were mentioned by some of our participants were “*security*” and “*stability*.” This is illustrated by a participant who shared, “*if you are on the path to recovery and you have to worry about a lot of things*, *like your next meal or you know*, *how you are gonna get through the night*, *that can push you in the opposite direction*. *So*, *security*, *knowing that you know*, *the things in your life are gonna be consistent… so you can focus on yourself*.”

## Discussion

In this study, we wanted to understand the concept of good health in the eyes of youth who experienced MHSU disorders and identify the factors that may prevent them from achieving good health. Our results provided their narrative of what health is to them. As shown in Figure 3, to our participants, health is embedded in the context of one’s environment, a consequence of the systemic and social influences. Health consisted of physical health, mental health, day to day functioning, and being in control of your own health condition. Systemic and social factors were factors that influenced the state of health. Recognition of these causal and mitigating factors and distinguishing them from the construct of health is important as the measurement of health and design of mental health services will not be accurate if these factors were not addressed.

Good health, in the eyes of our participants, was a continuum, which consisted of physical, mental, and social components along with a functional element when defining health. The concept that health is a continuum is not new, but our results highlighted the struggles with “sobriety” that participants faced and showed that the concept of health expands beyond the definition of health by WHO (30–32). Staying sober was difficult and something that had to be achieved to be considered “healthy.” Resilience and playing an active role in the management of their health condition were also recurring themes. When thinking about the evaluation of IYS programs, measures reflecting these outcomes should be included. Items reflecting daily functioning, self-image, and emotional health are common in many health-related quality of life (HRQL) measures like the Euroqol-5D (33). However, the struggles with “sobriety” are not often reflected in many generic health measures (31, 32). Looking physically healthy, like being free of scars and being a healthy weight, were important physical indicators associated with recovery from substance use. For evaluation of health outcomes in youths with MHSU disorders, items or outcomes reflecting recovery and coping with addiction should also be included.

The transition from adolescence to young adulthood is characterized by major social role changes and growing societal expectations. Having a MHSU disorder during this period could hamper life opportunities and shape quality of life in later life (34). For our participants with MHSU disorders, systematic and social factors were impacted their perception of health. The impact may vary from participant to participant but in general, the presence of these factors have been shown to mitigate the risk of early substance use and mental health issues in youths (35–37). This is aligned with previous studies with children and youths, emphasizing the importance of these factors (26, 38). Prevention, early intervention and increasing access to appropriate treatment resources are the best treatment strategies in youth MHSU care and a continuum of care, ranging from early intervention to crisis response, is needed for youths (39). Presently, while medications for substance use have been known to reduce substance use, access to these medications is limited for youths (40). Therefore, continued engagement and support of youths with MHSU disorders and ensuring access to health services are crucial in attaining a good health state. In addition, from a measurement perspective, it is important to tease out these factors to be able to understand and accurately measure outcomes that matter. This supports recent calls to redefine health, beyond the absence of disease and infirmity, and include meaningful domains that can inform health services and policy (3, 41).

The notion that health is more than just individual behavior and health habits is also supported by other frameworks of health and determinants of health such as the Wider Determinants of Health Model and the Mandala of Health model (42, 43). Both models depict how interactions with the environment affect health. The definition of health and the design of health services for youths must acknowledge and address the systemic and social factors that will influence their ability to achieve good health. In addition, often the conceptualization and measurement of health is focused on ill-health, however, when we recognize that health is a continuum, the focus can be shifted to the more favorable end of the continuum and health services can be focused on moving people toward “good health” (44). This shift in perspective is needed and we recommend actively involving the intended audience in designing timely interventions for this population (25, 26, 38, 45).

Lastly, our findings indicated that the path of recovery involved learning to manage their MHSU disorders. Recovery was a challenging path characterized by periods of ups and downs (46). Both mental health professionals and patients should be aware and prepared for setbacks on the path of recovery. Learning to manage their health condition, which will include both physical and mental health symptoms, is therefore an important element and should be included in the standard treatment for youths with MHSU disorders.

Strengths of this study include the involvement of participants throughout the iterative coding process. This engagement provided the opportunity for triangulation and ensured that results were representative of participants’ experience. The participation of a peer research partner in each phase of this project is another major strength of our study (47). This ensured that questions were appropriate, and results was understandable to the target population. Our peer research partner was also a PSW at the center, with the intention that having a trusted, established peer involved in facilitation would increase the comfort of participants.

There were also limitations that warrant discussion. Our sample consisted of a specific age group, 17–24 years old, with the majority being over age 19, and most participants had access and were receiving mental health services. This may limit the applicability of our findings to other populations. Future research is needed to explore definitions of health among diverse samples of youth experiencing varied health vulnerabilities and protective factors.

## Conclusion

This study contributes evidence that health in the context of MHSU disorders, is a multidimensional construct impacted by basic safety and security needs. MHSU services for youth should take a more holistic approach in treatment and address these basic needs and other social determinants of health as part of the treatment plan.

## Data availability statement

The datasets presented in this article are not readily available because the data are not publicly available due to restrictions, i.e., their containing information that could compromise the privacy of research participants. Requests to access the datasets should be directed to nikki.ow@ubc.ca.

## Ethics statement

The studies involving humans were approved by Providence Health Care Behavioral Research Ethics Board (#H17-00127). The studies were conducted in accordance with the local legislation and institutional requirements. Written informed consent for participation in this study was provided by the participants’ legal guardians/next of kin. Written informed consent was obtained from the individual(s), and minor(s)’ legal guardian/next of kin, for the publication of any potentially identifiable images or data included in this article.

## Author contributions

NO: Writing – review & editing, Writing – original draft, Visualization, Validation, Methodology, Formal analysis, Data curation, Conceptualization. RZ: Writing – review & editing, Writing – original draft, Data curation, Conceptualization. KT: Writing – review & editing, Supervision, Resources, Project administration, Funding acquisition. SM: Writing – review & editing, Supervision, Resources, Project administration, Funding acquisition. SB: Writing – review & editing, Supervision, Resources, Project administration, Methodology, Investigation, Funding acquisition, Conceptualization.
